# Prevalence of Smoking Various Tobacco Types in the Kazakhstani Adult Population in 2021: A Cross-Sectional Study

**DOI:** 10.3390/ijerph20021509

**Published:** 2023-01-13

**Authors:** Natalya Glushkova, Dariga Smailova, Zhanar Namazbayeva, Gulmira Mukasheva, Ayaulym Zhamakurova, Asylzhan Kuanyshkalieva, Indira K. Karibayeva, Almagul Kauysheva, Nurzhamal Otyzbayeva, Maksut Kulzhanov, Yuliya Semenova

**Affiliations:** 1 Department of Epidemiology, Evidence Based Medicine and Biostatistics, Kazakhstan’s Medical University “Kazakhstan School of Public Health”, Almaty 050000, Kazakhstan; 2 Department of Epidemiology, Biostatistics and Evidence Based Medicine, Al-Farabi Kazakh National University, Almaty 050040, Kazakhstan; 3 Asfendiyarov Kazakh National Medical University, Almaty 050040, Kazakhstan; 4 Department of Medicine, School of Medicine, Nazarbayev University, Astana 010000, Kazakhstan

**Keywords:** smoking, Kazakhstan, daily tobacco smoking prevalence

## Abstract

Tobacco use was the second-leading risk factor for death, accounting for 15.4% of total deaths in 2019. In 2019, 20.4% (2.7 million) of the adult population in Kazakhstan, 36.5% of men, and 6.0% of women smoked tobacco. A cross-sectional study of a random sample (n = 1201) was conducted between October and December 2021 in accordance with the STEPwise approach. The tobacco-use questions were focused on current and previous smoking status, initiation and duration of smoking, amount of tobacco use, exposure to secondhand smoke, and information related to quitting smoking. From 20.8% of smokers, 93.8% of men and 80.2% of women use tobacco products daily, χ^2^ = 10.983, *p*-score < 0.001. The earliest initiation of smoking was 6 years old. The prevalence of smoking tobacco products in Kazakhstan is 20.8%, which means that every fifth adult smokes. In addition, the proportion of smokers among men was 38.5%, and among women, it was 10.1%. A total of 93.8% of men and 80.2% of women smoked daily. The role of healthcare professionals in smoking prevention is very low, and only 16.9% of respondents have been advised to quit smoking in the last 12 months. New interventions for tobacco smoking prevention are urgently needed in Kazakhstan.

## 1. Introduction

Tobacco use is the second-leading risk factor for death, accounting for an 8.71 million loss of life worldwide (15.4% of total deaths) in 2019 [1]. It is believed that approximately 100 million people died in the 20th century, which often occurred in developed countries [2]. If current smoking trends continue, tobacco will kill an estimated 1 billion people by the end of the 21st century, with the majority of deaths occurring in low- and middle-income countries [3]. In fact, in 2019, approximately 77.5% (6 out of 7.69 million) of all tobacco-related deaths occurred in low- and middle-income countries [4].

Smoking is a known cause of cancer, cardiovascular disease, stroke, lung disease, diabetes mellitus, and other noncommunicable diseases (NCDs) [5]. In addition, it is one of the main preventable causes of premature death and disease worldwide [6]. Based on the WHO estimates, 1.1 billion people globally smoke [7], and Europe is the region with the highest prevalence of tobacco smoking [8]. In the EU, the highest smoking prevalence was found in Greece (42%), Bulgaria (38%), and Croatia (36%), while the lowest smoking abundance was observed in the Netherlands, followed by the United Kingdom (12%) and Sweden (7%) [9,10]. Almost 30.8 million adults in the United States currently smoke cigarettes, and more than 16 million Americans are living with smoking-related illnesses [11,12]. Tobacco-related disease costs (including health costs and lost productivity) for the global economy are more than $1 trillion per year, which is nearly 1.8% of the global gross domestic product (GDP). Smoking is a heavy economic burden worldwide, especially in Europe and North America where the tobacco epidemic is most developed [13].

Kazakhstan is the largest Central Asian state with one of the lowest population densities in the world. The population of Kazakhstan is relatively young, and approximately 50% of people are under the age of 30. The country implements a principle of equal gender rights, and thus, both sexes have equal access to education and equal business opportunities. However, women are still underrepresented in executive management positions [14]. The death rate is above the global average, and there is a striking discrepancy between life expectancy for men and women, which constitutes 67 and 77 years, respectively. This might be attributed to a higher prevalence of unhealthy lifestyle habits among men. Cardiovascular disease is the major cause of mortality and is also the main driver of premature mortality, followed by neoplasms, diabetes mellitus, and chronic pulmonary disease. The consumption of tobacco is among the modifiable risk factors for premature mortality that contributed to 16.4% of all deaths in 2019 [15].

To promote healthy lifestyles and combat tobacco consumption, the government of Kazakhstan drastically increased tobacco excise taxes in 2014, which resulted in a decline in smoking prevalence. According to two surveys, smoking prevalence among adults dropped by approximately 20% [16,17]. Although youth tobacco consumption remained unchanged between 2004 and 2009, it sharply declined in 2014. However, since the beginning of 2015, Kazakhstan has adopted a policy of moderate tax increases that failed to ensure significant tobacco consumption reductions [18].

The latest available data for 2019 indicate that 20.4% (2.7 million) of the adult population in Kazakhstan, 36.5% of men and 6.0% of women smoked tobacco. The average number of cigarettes smoked per day by daily smokers was 15.9 for men and 12.6 for women. The mean age of initiation among all those who had ever smoked tobacco was 17.7 years, with no dramatic differences by gender or place of residence. Next, 1.4% of adults over 15 reported using smokeless tobacco products. Overall, 11.4% of adults aged 15 and older working indoors were exposed to secondhand smoke at work (14.6% of men and 7.8% of women). Additionally, 9.1% of the adult population reported that they had been exposed to secondhand smoke at home. The prevalence of exposure to tobacco smoke at home was 12.2% among urban residents and 4.6% in rural areas [19].

As no data are available after 2019, there is a need for continuous monitoring of smoking prevalence in the country. A better understanding of smoking habits, types of tobacco products consumed, and age and sex differences will guide the formulation of evidence-based public health strategies targeted at a substantial reduction in tobacco consumption. Therefore, the aim of this study was to evaluate the prevalence of tobacco use in the adult population of both sexes residing in Kazakhstan.

## 2. Materials and Methods

### 2.1. Study Design and Setting

A cross-sectional survey of a random sample (n = 1201) was conducted between October 2021 and December 2021 in accordance with the WHO recommendations for the Control of Noncommunicable Diseases (NCDs). The standard STEPwise approach to surveillance (STEPS) was used, which is a standardized, internationally comparable tool that enables the collection, analysis, and dissemination of core information on NCDs. For study purposes, a validated version of the questionnaire in the Russian language was used [20]. A pilot study with 80 participants was undertaken to pretest the survey and ensure that it is culturally sensitive.

Two standardized steps of the STEPwise approach were utilized in data collection: (1) the structured demographic and medical history questionnaire, followed by (2) physical measurements. The target group was the adult population of the Zhambyl region, Kazakhstan (aged 18–69 years), who were able to undergo data collection procedures. The Zhambyl region was selected because it is a representative region of the country based on demographic structure and socioeconomic development [21]. The total sample consisted of 1201 healthy individuals of both sexes who attended local outpatient clinics for a routine check-up or accompanied family members. Systematic random sampling was applied to recruit a maximum of 20 individuals per day from each outpatient clinic.

### 2.2. Inclusion Criteria

- People aged 18 to 69 years.
- Willingness to provide informed consent.

### 2.3. Exclusion Criteria

- The population permanently residing in any residential institution, including social service institutions; hospitals or other health facilities; establishments owned by religious organizations; and those who are in detention houses, medical, and labor dispensaries or correctional institutions.
- Persons who do not have a permanent place of residence.

### 2.4. Data Collection

Data collectors met the survey participants between October 2021 and December 2021. After informed consent was received, the participants were interviewed in a privately designed place by means of face-to-face interviews that were carried out on the basis of a questionnaire on demographics and socioeconomic status, tobacco use, diet, physical activities, and self-reported medical history. The questions on tobacco use were focused on smoking status, both current and previous, as well as the initiation and duration of smoking, the amount of tobacco use, exposure to second-hand smoke, and information related to quitting smoking. More than 50% of data collectors were females to facilitate the disclosure of information by female participants.

### 2.5. Definition of Smoking

In this survey, the term “ever smoker” was applied to an individual who had ever smoked any tobacco products in his/her past. Meanwhile, “current smoker” was an individual who self-identified himself/herself as a smoker of any tobacco product at the time of the survey. This term applied both to regular and casual smokers. We also considered the number of currently used tobacco products. “Exposure to secondhand smoke” was also evaluated on the basis of self-assessment and included inhaling smoke from burning tobacco products. The term “smokers who tried to stop smoking” was used for those individuals who reported an attempt to quit smoking during the past year, irrespective of the type of tobacco smoked.

### 2.6. Statistical Analysis

All data were entered into an Excel spreadsheet and then exported to the SPSS version 24.0 for Windows. The type of data distribution was tested using the Kolmogorov-Smirnov test. Because the data distribution was normal, descriptive statistics were generated by computing the mean and the standard deviation (SD). Student’s *t*-test was applied to evaluate differences in means. Qualitative data are presented in absolute numbers and percentages. Pearson’s chi-square (χ^2^) test was used to evaluate differences in frequencies. The critical value was considered significant at *p* < 0.05.

## 3. Results

The general characteristics of the study population are presented in Table 1. The mean age of the study participants was 44 ± 14 years for males and 45 ± 13 years for females, which was statistically insignificant. There were significantly more single females (not married, divorced, or widowed) than males. Sex disaggregation of data was applied, as earlier research reported different rates of tobacco consumption in men and women in Kazakhstan [19].

The sociodemographic and physical characteristics of regular vs. nonregular smokers are presented in Table 2. There were significantly more regular smokers among males than among females: 72.0% and 28.0%, respectively. Additionally, regular smokers had insignificantly higher levels of systolic arterial pressure (126 vs. 120 mm Hg) and significantly higher levels of diastolic arterial pressure (86 vs. 80 mm Hg). The differences between other sociodemographic and physical parameters did not reach statistical significance.

For the variable “Do you currently smoke any tobacco products, such as cigarettes, cigarettes or pipes?” Of the 1201 respondents, 250 (20.8%) answered “Yes”, of which 174 (38.5%) were men and 76 (10.1%) were women, χ^2^ = 137.439, *p* < 0.001. Of these, 167 (93.8%) men and 65 (80.2%) women used tobacco products daily, χ^2^ = 10.983, *p*-score < 0.001 (Table 3).

Of the respondents, they smoked for the first time at the age of 19 (the maximum value is 50 years, the minimum is 6 years), of which men: the average age is 18 years (max-37 years), women: the average age is 22 years (max 50 years old), T = −4.983, *p*-value < 0.001. According to the results of the survey “How many manufactured cigarettes, on average, do you smoke per week?”, the average number was 81 cigarettes, max-210, min-4. Of these, men smoked an average of 93 cigarettes (max-210) per week, and women smoked an average of 53 cigarettes (max-140) per week (Table 4).

Of the respondents who currently use any tobacco products, 123 respondents answered “yes” to the question “Have you tried to quit smoking in the last 12 months?”, of which 89 (51.1%) were men and 34 (44.7%) were women.

Only 203 (16.9%) respondents were advised to stop smoking during any visit to a doctor or other health worker in the last 12 months, of which 116 (25.7%) were men and 87 (11.60%) were women. Of the respondents, only 176 (18.3%) respondents smoked tobacco products in the past, 115 (41.1%) men and 61 (8.9%) women, χ^2^ = 137.346, *p* = <0.001. Of these, 100 (80.6%) men and 50 (64.1%) women smoked daily in the past, χ^2^ = 6.855, *p* = 0.009 (Table 5).

Out of 1201 respondents, only 8 (0.7%) respondents answered that they currently use any nonsmoking (smokeless) tobacco products (snuff and chewing tobacco, nasvay), χ^2^ = 13.346, *p* < 0.001. Among those who answered “yes”, all were men. Of these, 7 (58.3%) currently use any nonsmoking (smokeless) tobacco products daily, χ^2^ = 4.958, *p* = 0.026. Of the respondents, 9 (2%) men and 3 (0.4%) women had used nonsmoking (smokeless) tobacco products (snuff, nasvay) in the past (χ^2^ = 7.431, *p* = 0.006). Of these, 3 (23.1%) used smokeless tobacco products daily. To the question “In the past 30 days, has anyone smoked in your home?” Of the 1201 respondents, 372 (31.0%) answered “yes”, including 123 (27.2%) men and 249 (33.2%) women, χ^2^ = 4.797, *p* = 0.29. Of the 1201 respondents, 131 (29%) men and 105 (14%) women answered “yes” to the question “During the past 30 days, has anyone smoked in the room where you work (in the building, in the work area or in other offices?”, χ^2^ = 41.025, *p* < 0.001 (Table 6).

## 4. Discussion

This cross-sectional survey was designed to provide a population-based prevalence estimate of smoking and related risk factors in the adult population of the Zhambyl region on the basis of the 2021 WHO-STEPS survey. Our results show that the rate of current tobacco use among the survey participants was 20.8%. Earlier reports indicate that the national prevalence of cigarette smoking was 22.4% in 2014 [22] and 21.5% in 2019 [19], which is higher than this finding. Apart from providing estimates on the prevalence of smoking, this research contributes to the existing literature in a variety of ways. In general, there is a paucity of data on smoking habits in the population of Kazakhstan. During the period 2010–2020, the data were generated by two GATS surveys [16,19] and a local study [17] which did not follow the international methodology. The present study is the first to describe the physical characteristics of participants by the pattern of smoking. It details information about different types of heated and smokeless tobacco products consumed, and gives insights into the tobacco cessation experience.

Based on the WHO estimates, as many as one-fifth (22%) of the global adult population currently uses tobacco products, and the vast majority of them do this on a daily basis. The prevalence of smoking varies greatly between men and women: 42% vs. 5% [23]. For adolescents, the 2014 Global Youth Survey reported smoking in 4% of boys and 2% of girls aged 13–15 years. In Kazakhstan, boys smoke more often than girls, but this trend is increasing among girls [24]. According to the WHO projections, which were based on the 2014 level of adult smoking, as many as 50% (1.4 million) of the 2.8 million smokers in Kazakhstan are likely to die prematurely, and the lack of strong policies plays a significant role [23].

According to our findings, men start smoking at a younger age and consume more cigarettes than women. Similar findings were reported by studies conducted in other countries of the former Soviet Union. Shkolnikov et al. reported significantly lower rates of smoking among Russian women than among their counterparts in the majority of Western countries. The mean age of smoking initiation was 16.6 years for men and 18.6 years for women [25]. In Ukraine, as many as 70% of men start smoking before the age of 20 in comparison with 20% of women [26]. Internationally, men begin smoking earlier than women, but there is a large regional variation in the size of between-sex differences. In general, this difference is almost negligible in countries with high levels of income and reaches 8 years in East and Southeast Asia. It is a common observation that countries with large between-sex differences in the prevalence of smoking also have large differences in the age at smoking initiation [27]. 

As our data demonstrate, more than 50% of men and nearly 45% of women tried to quit smoking in the last 12 months. It is not easy to achieve high rates of smoking cessation, especially for established smokers. The onset of smoking commonly falls at a young age, when people are very susceptible to addictions [28]. Therefore, it is important to minimize the number of people starting to smoke through effective strategies incorporating different sectors of society, such as education, nongovernmental organizations, religious communities, and the private sector [29]. 

According to our findings, men had significantly higher rates of tobacco use. This was consistent with earlier data, including the Global Adult Tobacco Survey (2019) and the WHO report (2019). According to the results, 38.5% and 10.1% of men and women, respectively, consume tobacco products. A systematic review of tobacco smoking in various studies has shown that men use tobacco products more often than women do [6,30,31,32]. The higher prevalence of tobacco use among men might be associated with the peculiarities of sex roles in society. Thus, men tend to have greater social power, which can lead to health policies prohibiting tobacco consumption by women, and this is likely to be the major cause of gender differences in tobacco use [6].

The comparison with other countries is interesting. In China, the rates of adult ever smokers and current smokers among men were 62.4% and 54.0%, respectively. At the same time, these rates were 3.4% and 0.8% among women [33]. Furthermore, in 2016, the prevalence of current adult cigarette smokers among US men was 17.5% compared with 13.5% in women [34]. Considering the significantly higher prevalence of smoking among men in our study, health promotion campaigns should consider this and focus, in particular, on the male population. From 2000 to 2018, the number of men using tobacco in the world increased every year. This number peaked in 2018 and amounted to 109.3 million tobacco users. The number of users is expected to fall to 1.087 billion in 2025 if countries continue their current tobacco control efforts. In the period 2000–2015, the number of women using tobacco in all regions decreased, and it is expected that this number will continue until 2025. It is estimated that there were 100 million fewer women using tobacco (244 million) in 2018 than in 2000 (346 million). This number is expected to decrease to approximately 212 million by 2025 [35].

The overall prevalence of tobacco use in Kazakhstan in 2019 did not undergo any significant changes compared to 2014 [16]. According to our study, tobacco consumption decreased among men from 43.4% to 38.5% but increased among women from 4.5% to 10.1%. The increase in tobacco use among women might be attributed to the growing popularity of vaping devices that are perceived to be safer than other tobacco products [27]. Earlier research indicated that 1.2% of adults smoked hookah on a regular basis [19], which is generally consistent with our findings: approximately 1.7% of adults smoked hookah every week, and approximately 0.5% smoked it on a daily basis. There is a declining trend in the number of cigarettes smoked every day. According to a previous study, the average number of daily cigarettes smoked was 15.9 for men and 12.9 for women [19], while in our study, these numbers equaled 14 and 9, respectively. Additionally, in 2019, 1.4% of current tobacco users used smokeless products, of whom 2.7% were men and 0.1% were women [19]. In this study, nearly 1.0% of people reported current usage of smokeless tobacco products: 2.1% of men and 0.4% of women. In Kazakhstan, as in many other parts of the world, the causes of smoking are complex and interrelated. People smoke because it helps to relieve stress, gives pleasure, and is socially acceptable in many situations. Additionally, smoking has been affordable since the beginning of 2015, and the government has been practicing a moderate approach to excising tax increases [18]. 

The measures taken by Kazakhstan to comply with a partial ban on smoking in certain closed public places led to a significant reduction in exposure to tobacco smoke in closed public places, from 13.8% to 9.1% at home, from 19.1% to 11.4% in the workplace, and from 18.1% to 9.0% in public transport [16]. Raising tobacco taxes is another intervention that might effectively reduce tobacco consumption.

The study has limitations because smoking-related questions are sensitive for women in Kazakhstan, and many of them may avoid answering true questions about this habit.

## 5. Conclusions

The prevalence of smoking tobacco products in Kazakhstan is 20.8%, which means that every fifth adult smokes. In addition, the proportion of smokers among men was 38.5%, and among women, it was 10.1%. A total of 93.8% of men and 80.2% of women smoked daily. The role of healthcare professionals in smoking prevention is very low, and only 16.9% of respondents have been advised to stop smoking in the last 12 months. New interventions for tobacco smoking prevention are urgently needed in Kazakhstan.

## Figures and Tables

**Table 1:** General sociodemographic characteristics of study participants, n = 1201.

Variables		Gender						Test of Difference	
		Male		Female		Total		χ^2^	*p*-Value
		n	%	n	%	n	%		
Age, years, mean, and standard deviation *		44 ± 14		45 ± 13		45 ± 14		1.82	0.178
Education, years, mean, and standard deviation *		14	3	14	3	14	3	5.04	0.025
Education level	Completed primary education (4 grades)	2	0.4	2	0.3	4	0.3	8.335	0.080
	Completed secondary education (9 grades)	50	11.1	70	9.4	120	10.0		
	Completed secondary education (11 grades)	152	33.7	295	39.4	447	37.3		
	Higher	231	51.2	369	49.3	600	50.0		
	Master/Doctoral	16	3.5	12	1.6	28	2.3		
Ethnicity	Kazakh	248	54.9	383	51.1	631	52.5	13.031	0.043
	Russian	86	19.0	198	26.4	284	23.6		
	Uzbek	36	8.0	52	6.9	88	7.3		
	Ukraine	9	2.0	10	1.3	19	1.6		
	Uygur	2	0.4	0	0.0	2	0.2		
	Tatar	11	2.4	22	2.9	33	2.7		
	Other	60	13.3	84	11.2	144	12.0		
Family status	Single, not married	78	17.3	120	16.0	198	16.5	52.300	<0.001
	Married	352	77.9	491	65.6	843	70.2		
	Married/married but living separately	1	0.2	5	0.7	6	0.5		
	Divorced	13	2.9	73	9.7	86	7.2		
	Widower/widow	4	0.9	57	7.6	61	5.1		
	Civil marriage	4	0.9	3	0.4	7	0.6		
Employment status	State employee	50	11.1	73	9.8	123	10.3	127.381	<0.001
	Private sector worker	156	34.7	209	27.9	365	30.5		
	Budget employee	44	9.8	119	15.9	163	13.6		
	Entrepreneur	86	19.2	75	10.0	161	13.5		
	Agricultural worker	10	2.2	10	1.3	20	1.7		
	Student	10	2.2	15	2.0	25	2.1		
	A housewife	4	0.9	128	17.1	132	11.0		
	Pensioner	51	11.4	104	13.9	155	12.9		
	Unemployed (able to work)	35	7.8	12	1.6	47	3.9		
	Unemployed (unable to work)	3	0.7	3	0.4	6	0.5		

*—*t*-test.

**Table 2:** Sociodemographic and physical characteristics by pattern of smoking, n = 251.

Variables		Regular Smokers		Nonregular Smokers		Test of Difference	
		n	%	n	%	χ^2^	*p*-Value
Gender	Male	167	72.0	8	42.1	7.426	0.006
	Female	65	28.0	11	57.9		
Age, years, mean, and standard deviation *		44	12	40	13	1.533	0.126
Education, years, mean, and standard deviation *		13	3	13	3	0.292	0.770
Education level	Completed primary education (4 grades)	1	0.4	0	0.0	2.900	0.575
	Completed secondary education (9 grades)	31	13.4	4	21.1		
	Completed secondary education (11 grades)	80	34.6	4	21.1		
	Higher	115	49.8	10	52.6		
	Master/Doctoral	4	1.7	1	5.3		
Ethnicity	Kazakh	114	49.1	12	63.2	2.594	0.762
	Russian	59	25.4	4	21.1		
	Uzbek	14	6.0	1	5.3		
	Ukraine	7	3.0	0	0.0		
	Uygur	0	0.0	0	0.0		
	Tatar	7	3.0	1	5.3		
	Other	31	13.4	1	5.3		
Family status	Single, not married	42	18.1	3	15.8	1.289	0.936
	Married	161	69.4	15	78.9		
	Married/married but living separately	2	0.9	0	0.0		
	Divorced	19	8.2	1	5.3		
	Widower/widow	6	2.6	0	0.0		
	Civil marriage	2	0.9	0	0.0		
Employment status	State employee	22	9.5	1	5.3	9.481	0.303
	Private sector worker	88	38.1	6	31.6		
	Budget employee	19	8.2	2	10.5		
	Entrepreneur	49	21.2	6	31.6		
	Agricultural worker	5	2.2	0	0.0		
	Student	1	0.4	1	5.3		
	A housewife	12	5.2	2	10.5		
	Pensioner	18	7.8	1	5.3		
	Unemployed (able to work)	17	7.4	0	0.0		
	Unemployed (unable to work)	0	0.0	0	0.0		
BMI groups	≤24.9	4	1.8	0	0.0	0.870	0.647
	25–29.9	74	33.3	8	42.1		
	>30	144	64.9	11	57.9		
Waist circumference, cm		92	30	82	14	1.500	0.135
Hip circumference, cm		105	15	104	14	0.207	0.836
Systolic arterial pressure, mm Hg		126	17	120	16	1.549	0.123
Diastolic arterial pressure, mm Hg		86	11	80	10	2.265	0.024
Heart rate		78	11	76	10	0.841	0.401
Pregnancy (for females)	Yes	1	1.5	0	0.0	0.171	0.679
	No	64	98.5	11	100.0		

*—*t*-test.

**Table 3:** Smoking status, n = 1201.

Variables		Gender						Test of Difference	
		Male		Female		Total		χ^2^	*p*-Value
		n	%	n	%	n	%		
Current tobacco smokers	Yes	174	38.5	76	10.1	250	20.8	137.439	<0.001
	No	278	61.5	673	89.9	951	79.2		
Daily smokers of any tobacco	Yes	167	93.8	65	80.2	232	89.6	10.983	<0.001
	No	11	6.2	16	19.8	27	10.4		

**Table 4:** Smoking status characteristics, n = 1201.

Variables	Gender						Test of Difference	
	Male		Female		Total		*t* Test	*p*-Value
	Mean	SD *	Mean	SD *	Mean	SD *		
At what age did you smoke for the first time?	18	4	22	8	19	6	−4.983	<0.001
How many manufactured cigarettes, on average, do you smoke daily?	14	7	9	6	12	7	4.976	0
How many manufactured cigarettes do you smoke per week on average?	93	46	53	43	81	48	5.549	<0.001
How many hand-rolled cigarettes do you smoke daily on average?	2	1			2	1	NA	NA
How many hand-rolled cigarettes do you smoke per week on average?	10	4			10	4	NA	NA
How many pipes filled with tobacco do you smoke daily on average?			5		5		NA	NA
How many pipes filled with tobacco do you smoke per week on average?							NA	NA
How many hookah sessions (once charged hookah), on average, do you smoke daily?	8	11	2		6	9	0.459	0.691
How many hookah sessions (once charged hookah), on average, do you smoke per week?	20	49			20	49	NA	NA
How many other tobacco products, on average, do you smoke daily?	5	5	10		7	4	−0.896	0.465
How many other tobacco products do you smoke on average per week?	11	13	3	1	7	9	0.943	0.445

* SD—standard deviation.

**Table 5:** Characteristics of tobacco cessation experience, n = 1201.

Variables		Gender						Test of Difference	
		Male		Female		Total		χ^2^	*p*-Value
		n	%	n	%	n	%		
Has your health worker advised you to stop using tobacco products or not to start at all?	Yes	116	25.70	87	11.60	203	16.90	39.608	<0.001
	No	336	74.30	662	88.40	998	83.10		
Have you tried to quit smoking in the last 12 months?	Yes	89	51.10	34	44.70	123	49.20	0.87	0.351
	No	85	48.90	42	55.30	127	50.80		
Have you smoked any tobacco products in the past?	Yes	115	41.10	61	8.90	176	18.30	137.346	<0.001
	No	165	58.90	622	91.10	787	81.70		
Have you smoked daily in the past?	Yes	100	80.60	50	64.10	150	74.30	6.855	0.009
	No	24	19.40	28	35.90	52	25.70		
How old were you when you quit smoking?		36	12	32	10	34	12	1.95	0.05

**Table 6:** Characteristics of smoking status of smokeless tobacco products, n = 1201.

Variables		Gender						Test of Difference	
		Male		Female		Total		χ^2^	*p*-Value
		n	%	n	%	n	%		
Do you currently use any nonsmoking (smokeless) tobacco products, such as snuff and chewing tobacco, nasvay?	Yes	8	1.8	0	0.0	8	0.7	13.346	<0.001
	No	444	98.2	749	100.0	1193	99.3		
Do you currently use nonsmoking (smokeless) tobacco products daily?	Yes	7	58.3	0	0.0	7	41.2	4.958	0.026
	No	5	41.7	5	100.0	10	58.8		
How many times per day, on average, do you consume wet snuff, nasvay?		3	1			3	1	NA	NA
How many times per week, on average, do you consume wet snuff?		17	13			17	13	NA	NA
Do you use nonsmoking (smokeless) tobacco products, such as snuff, nasvay?	Yes	9	2.0	3	0.4	12	1.0	7.431	0.006
	No	434	98.0	746	99.6	1180	99.0		
In the past, did you use nonsmoking (smokeless) tobacco products such as snuff, tobacco, nasvay daily?	Yes	3	23.1	0	0.0	3	16.7	1.385	0.239
	No	10	76.9	5	100.0	15	83.3		
Has anyone in your home smoked in the last 30 days?	Yes	123	27.2	249	33.2	372	31.0	4.797	0.029
	No	329	72.8	500	66.8	829	69.0		
In the past 30 days, has anyone smoked in the area where you work (in the building, work area, or other office space)?	Yes	131	29.0	105	14.0	236	19.7	41.025	<0.001
	No	269	59.5	556	74.2	825	68.7		
	I do not work indoors	52	11.5	88	11.7	140	11.7		

## Data Availability

The datasets generated during and/or analyzed during the current study are available from the corresponding author on reasonable request.
